# Amyloid-beta–copper interaction studied by simultaneous nitrogen K and copper L_2,3__-_edge soft X-ray absorption spectroscopy

**DOI:** 10.1016/j.isci.2021.103465

**Published:** 2021-11-16

**Authors:** Jinghui Luo, Hongzhi Wang, Jinming Wu, Vladyslav Romankov, Niéli Daffé, Jan Dreiser

**Affiliations:** 1 Department of Biology and Chemistry, Paul Scherrer Institute, 5232 Villigen PSI, Switzerland; 2 Swiss Light Source, Paul Scherrer Institute, 5232 Villigen PSI, Switzerland

**Keywords:** Organometallic chemistry, Physical chemistry, Biophysical chemistry, Biochemistry

## Abstract

We study the interaction between amyloid β (Aβ) peptides and Cu and Zn metal ions by using soft X-ray absorption spectroscopy. The spectral features of the peptides and Cu are simultaneously characterized by recording spectra at the N K-edge and at the Cu L_2,3__-_edges. In the presence of the peptides, the Cu L_2,3__-_edge shows a fingerprint of monovalent Cu(I), caused by the interaction with the peptides. The appearance of Cu(I) is less significant at an acidic pH than at a basic pH. Furthermore, aggregation leads to a smaller signature of Cu(I). N K-edge spectra reveal that Cu and Zn ions exhibit a different coordination with the nitrogen atoms in the peptides. This suggests different roles of Cu and Zn in the peptide aggregation. Our work provides physical insight into the participation of the metal ions and Aβ in the toxic reactive oxygen species formation.

## Highlights

• Amyloid-beta–copper interaction leads to distinct X-ray spectroscopic signatures
• After interaction monovalent copper(I) is found
• The X-ray signatures strongly depend on the pH and incubation conditions

## Graphical abstract

**Figure:**
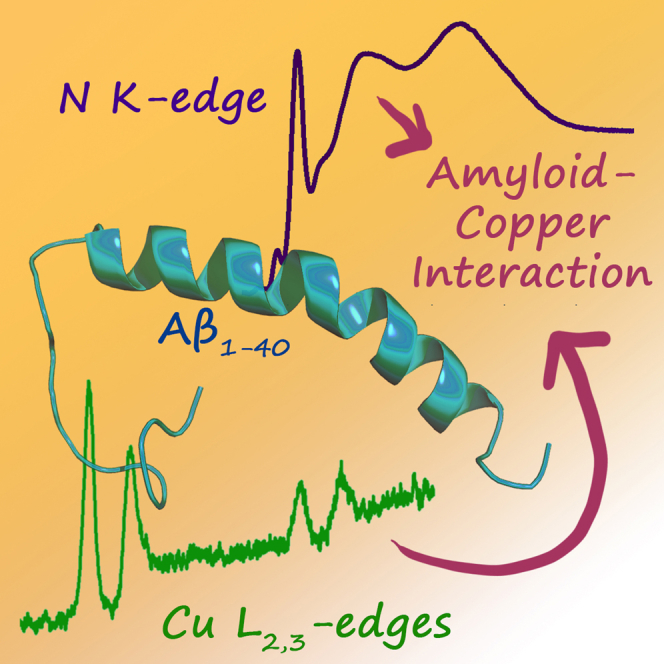

## Introduction

Alzheimer's disease (AD) is the most common form of progressively neurodegenerative diseases and poses an increasing burden for our aging societies. The disease is associated with the loss of neuronal connections and manifested by amyloid β (Aβ) fibril plaques and Tau neurofibrillary tangles in the brain (Ittner and Götz, 2011). Aβ is cleaved from intramembrane proteolytic processing of amyloid precursor protein (APP) by β-/γ-secretase (Selkoe and Hardy, 2016). The Aβ peptides may aggregate into transient and on-pathway oligomers, and eventually deposit as insoluble fibrils within the plaques (Luo et al., 2014) (Wu et al., 2020, 2021). Among these aggregates, on-pathway oligomers are assumed in many studies to induce the neuronal dysfunction (Glabe, 2008). Alternatively, the peptides can be stabilized as the off-pathway oligomers (Ehrnhoefer et al., 2008) with which the preformed fibril seeds do not promote fibril formation (Dear et al., 2020). However, cellular toxicity cannot be distinguished from the on- or off-pathway identity (Dear et al., 2020). In addition to the oligomer toxicity, it has been suggested that the concentration of metal ions, like copper, is increased by up to 5.7 times in the plaques of AD brains as compared with the healthy ones (Dong et al., 2003). Copper ions catalyze the formation of toxic reactive oxygen species (ROS) and cause damage to the surrounding brain tissue (Pithadia and Lim, 2012). The copper homeostasis imbalance influences Aβ toxicity, aggregation, and other intracellular processes (Kenche and Barnham, 2011; Maynard et al., 2005).

Over the past decade, a number of biophysical methods have allowed to characterize the interaction and coordination chemistry between Aβ and copper. In combination with density functional theory, X-ray absorption spectroscopy (XAS) studies suggested a six-coordinate (3N3O) geometry with copper in the complexes of Aβ_1-16_-Cu at pH 7.4 where residues His6, His13, His14, Glu11, or/and Asp1, and axial water are involved (Streltsov et al., 2008). At different pHs, the Aβ_1-16_-Cu complexes are prone to the formation of the 3N1O coordination sphere (Dorlet et al., 2009; Drew et al., 2009a,2009b; Trujano-ortiz and Quintanar, 2015). At pH 6.3–6.9, the copper coordination sphere is contributed by three amino acids from the Aβ_1-16_ peptide, His6, His13/His14, and Asp1. At pH 8, copper is coordinated with four amino acids, His6, His13, His14, and Ala2 (Drew et al., 2009a, 2009b). The latter 3N1O coordination sphere of copper with three nitrogens and one oxygen was confirmed by homology modeling techniques with quantum mechanics-based approaches (Alí-Torres et al., 2011). The Faller and Hureau lab also found two components of the Aβ1–16 and Cu(II) interactions at pH 6.5 and pH 9.0. Cu(II) binds to the carbonyl from the amide bond of Asp1–Ala2 and the imidazole nitrogens from His6 and His13/His14, at pH 6.5 (Atrián-Blasco et al., 2017). In the second component, the nitrogen atom of the Asp1–Ala2 amide bond binds to Cu(II) after deprotonation at pH 9.0 (Dorlet et al., 2009)(Atrián-Blasco et al., 2017). As for Zn(II), the N-terminal amine of the peptide does not coordinate to the metal ion at a physiological pH (Atrián-Blasco et al., 2017). Besides the mononuclear Cu(II) site, a binuclear Cu(II) site has also been proposed with a deprotonated histidine to bridge two Cu(II) ions (Karr et al., 2004). These suggest that the Aβ-Cu coordination sphere as well as its redox behavior could be determined by the pH. The understanding of the coordination environment of Cu-Aβ gives a mechanistic insight into the disease-related ROS production and provides an important perspective for the rational design of new chelators in the development of therapeutics against AD.

Despite the characterization of Cu surrounded by Aβ residues by techniques such as EPR (Electron paramagnetic resonance) (Dorlet et al., 2009; Posadas et al., 2021),NMR (Nuclear magnetic resonance) (Tiiman et al., 2016) (Luo et al., 2013), or hard X-rays (Shearer et al., 2010), the reduced state of Cu remains to be explored upon binding to Aβ or other amyloid proteins. Although EPR allows to detect Cu(II) with unpaired electrons, this technique does not directly give the properties of Cu(I) and nitrogen. NMR is only able to directly observe the properties of peptides rather than the ones of copper. Furthermore, the simultaneous investigation of Cu and the peptide is impossible using hard X-rays because of the absence of any nitrogen resonant excitations in the hard X-ray regime. XAS in the soft X-ray range employing photons of a few hundreds of eV up to 2 keV allows to study in an element specific way the spectroscopic signatures of Cu and N, which depend on their chemical environments (Kvashnina et al., 2009) (Shimizu et al., 2001) (Leinweber et al., 2007). More specifically, this involves excitations at photon energies around 930 … 950 eV reaching from the Cu 2p to the Cu 3d orbitals, the so-called Cu L_2,3_-edges thus probing empty states (holes) in the 3d shell. In the case of N 1s → 2p, excitations are probed at photon energies of 400 … 420 eV, which are termed the N K-edge. Importantly, soft XAS allows studying both Cu and N under exactly the same conditions in the same sample, which makes it a unique tool for the investigation of the interaction of peptides with transition metals. Here, we implement soft XAS to investigate the Cu L_2,3_-edges and the nitrogen K-edge in the complex of Aβ_1−40_ and Cu/Zn under various pH values and incubation conditions.

## Results

**Figure 1:**
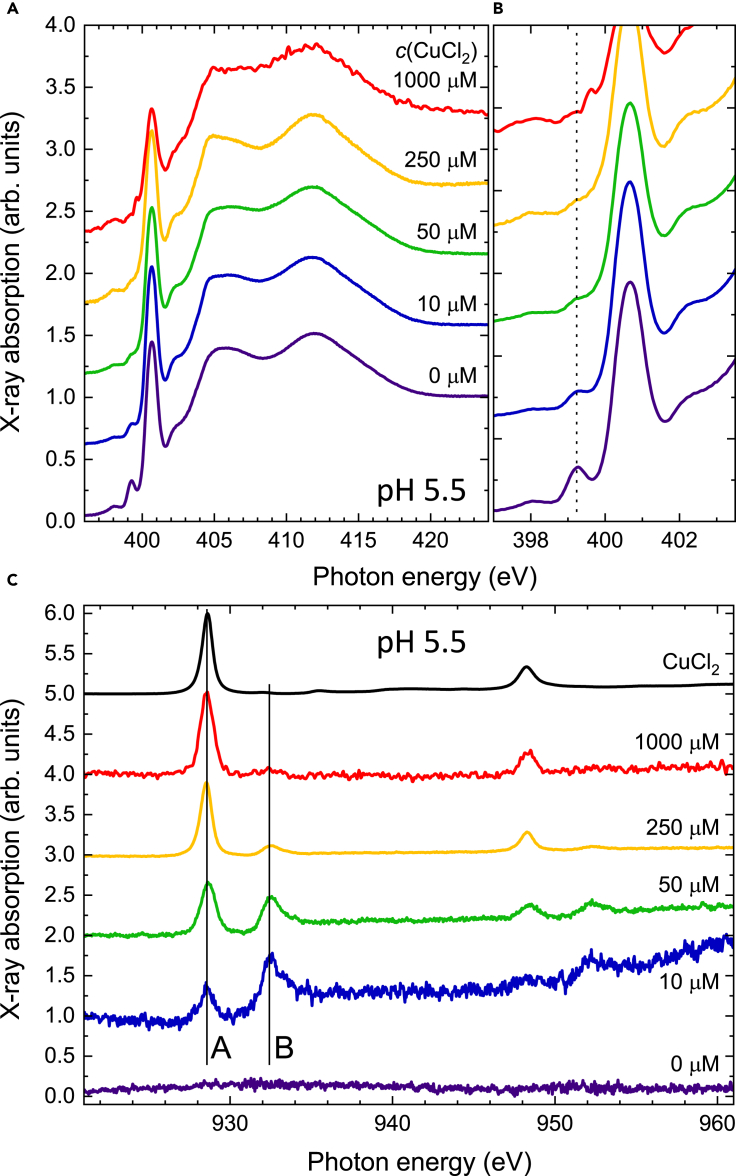
Effect of the concentration of CuCl_2_ mixed with Aβ_1–40_ at pH 5.5 X-ray absorption spectra recorded (A and B) at the nitrogen K-edge and (C) at the Cu L_2,3_-edges on 50 μM Aβ_1–40_ mixed with CuCl_2_ solutions of different concentrations at pH 5.5. 200 μL of sample was prepared with 50 μM Aβ_1–40_ and 20 mMKPi buffer, pH 5.5, in the presence of 0, 10, 50, 250, or 1000 μM CuCl_2_, and then 50 μL of each sample was deposited on a silicon dioxide wafer and immediately dried under nitrogen gas for recording X-ray absorption spectra. Vertical dashed and solid lines in (B) and (C) follow the features discussed in the main text.

Figure 1 displays X-ray spectra recorded on Aβ_1-__40_ samples prepared with different concentrations of CuCl_2_ at an acidic pH of 5.5. The N K-edge spectra can be separated into a π^∗^ excitation range at energies below ∼403 eV and a σ^∗^ one at higher energies. The π^∗^ excitation range includes a strong sharp peak at 400.6 eV and a smaller pre-peak feature located at a photon energy of 399.3 eV. The pre-peak feature is strongest without CuCl_2_, and upon increasing the CuCl_2_ concentration, it gradually disappears. This is clearly visible in the zoom shown in Figure 1B. The pre-peak feature of 50 μM Aβ is completely eliminated in the presence of 50 μM Cu(II). This is in agreement with a 1:1 reaction stoichiometric ratio of Cu(II) to Aβ (Atrián-Blasco et al., 2017). The spectrum of the 1000 μM sample (incubated for 1 day at 30°C) has a slightly lower intensity of the π^∗^ peak and a different pre-peak feature that could originate from the structural changes because of the different aggregation pathway in the presence of the concentrated Cu(II) ions (Han et al., 2021; Mold et al., 2013; Viles, 2012). At a high stoichiometric ratio of Cu(II) to Aβ, Cu(II) precipitates Aβ_42_ as amorphous deposits (Mold et al., 2013; Viles, 2012). Given our observations, we ascribe the loss of the pre-peak feature at 399.3 eV to the interaction of Aβ_1-40_ with Cu(II) ions from CuCl_2_.

The corresponding Cu L-edge spectra normalized to the total area of the peaks are plotted in Figure 1C. While peak **A** at an energy of 928.5 eV can be attributed to the excitation of Cu(II) ions exhibiting a 3d^9^ electronic configuration, for example, in small non-reacted clusters of CuCl_2_, a new species is observed giving rise to peak **B** at a photon energy of 932.5 eV. We attribute this peak to the Cu ions, which can be reduced to Cu(I) upon oxidation of the Aβ peptides possibly through the N-terminal residues, Asp1, Ala2, His6, His 13, and His14 (Trujano-ortiz and Quintanar, 2015). This is supported by the change of the spectral weight across the concentration series. The remarkably large splitting of ∼4 eV between peaks **A** and **B** excludes that **B** is related to Cu_2_O, where a splitting of ∼2.5 eV would be expected (Grioni et al., 1992). Furthermore, the comparison with the reference measurement on Cu(II)-phthalocyanine shown in the SI and with the literature allows us to exclude that peak **B** originates from Cu(II) or even Cu(III) species (Shimizu et al., 2001). The latter two species display ligand-field and oxidation-state induced peak shifts, yet these shifts occur within a spread of ∼2 eV below and ∼1 eV above the L_3__-_edge of the Cu(II)-phthalocyanine reference. In line with literature reports, peak **B** corresponds to the presence of a 3d^10^ configuration in monovalent Cu(I). Formally, in an isotropic environment the strong dipole allowed 2p→3d absorption would be completely suppressed because of the full 3d shell; however, in the presence of the Aβ_1–40_ peptide offering nitrogen ligands, the hybridization of the Cu 3d orbitals with Cu 4sp and with the ligand orbitals is possible (Hatsui et al., 2004). The transition energy is, however, significantly higher because of the much lower excitonic downshift as compared to Cu(II) (Grioni et al., 1992). Hence, we conclude that there is a significant fraction of monovalent Cu(I) interacting with the Aβ_1-40_ peptide. This is further corroborated by an X-ray magnetic circular dichroism measurement described in the supplemental information (Figure S1). Note that it is difficult to quantify the exact ratio of Cu(I) vs Cu(II) species because the X-ray absorption cross section of Cu(I) is expected to be lower owing to the smaller amount of 3d holes. This implies that at equal areas of peaks **A** and **B** there is more Cu(I) than Cu(II) present in the probed volume.

**Figure 2:**
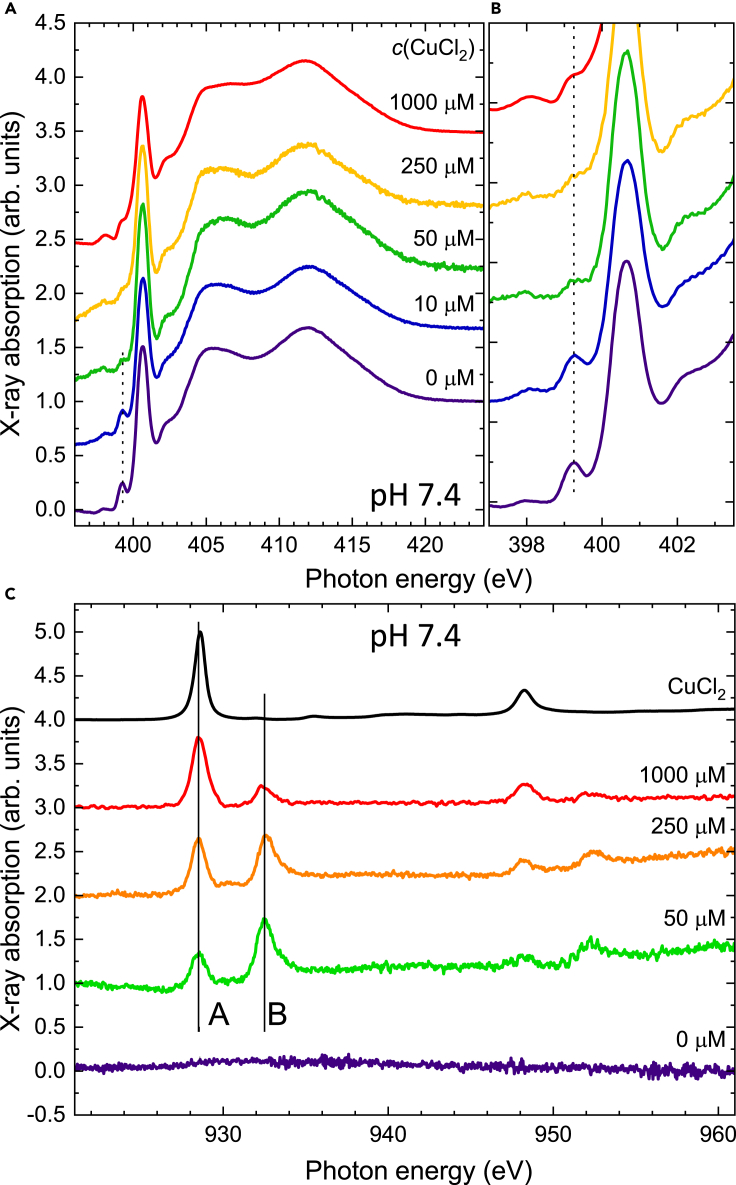
Effect of the concentration of CuCl_2_ mixed with Aβ_1–40_ at pH 7.4 X-ray absorption spectra recorded (A and B) at the nitrogen K-edge and (C) at the Cu L_2,3_-edges on Aβ_1–40_ mixed with CuCl_2_ solutions of different concentrations at pH 7.4. 200 μL of sample was prepared with 50 μM Aβ_1–40_ and 20 mMKPi buffer, pH 7.4, in the presence of 0, 10, 50, 250, or 1000 μM CuCl_2_, and then 50 μL of each sample was deposited on a silicon dioxide wafer and immediately dried under nitrogen gas for recording X-ray absorption spectra. Vertical lines in (C) follow peaks **A** and **B** discussed in the main text.

The analogous X-ray spectra of samples prepared at a neutral pH of 7.4 are shown in Figure 2. An inspection of the N K-edge spectra reveals that the evolution of the pre-peak feature is different in that it disappears only at a concentration of 250 μM whereas at the lower pH it is already absent at a concentration of about 50 μM. This striking behavior is also reflected in the Cu L-edge spectra where the equal height of the peaks of inorganically bound Cu(II) (peak **A**) and the one bound to the peptide (**B**) is reached at the concentrations of 50 μM (pH 5.5) and 250 μM (pH 7.4), respectively. The pH dependence of the pre-peak disappearance can be associated with the reduction to Cu(I). At pH 5.5, the peptides are prone to protonation with histidines with a more efficient redox cycling with Cu(II) than that of pH 7.4. The low pH at 5.5 is assumed as the acidosis, associated with inflammatory processes in AD and the elevated neurotoxicity of Aβ (Ghalebani et al., 2012). The acidosis may further explain the lower stoichiometric ratio between Cu and Aβ at a lower pH in our studies.

To characterize the dependence of the absorption spectra on the Aβ_1–40_ aggregation, we incubated part of the samples for 4 h at room temperature and for 1 day at 30°C (Broersen et al., 2011). In Table S1, the peak **B**/**A** ratios are given for different preparation conditions of Aβ_1-40_ and CuCl_2_ concentrations. At pH 5.5, the peak **B** of the Cu spectra seems less visible than at pH 7.4 in the presence of 4-h incubated and 24-h incubated Aβ_1-40_ aggregate, possibly representing small aggregates and insoluble large aggregates(Luo et al., 2014)(Broersen et al., 2011), respectively, indicative of the larger Aβ_1-40_-Cu aggregates formation from the acidosis. Apart from the trend of stronger Aβ_1-40_-Cu interaction at a lower pH, this also points to a dependence of the **B**/**A** ratio on the incubation time and temperature, which suggests the conversion of Cu(I) toward Cu(II) during the incubation process. It could also be due to structural modifications in the aggregation of the Aβ_1-40_ peptide and the buried Cu sites after incubation such that they are not detected anymore with the surface-sensitive total electron yield detection applied here.

**Figure 3:**
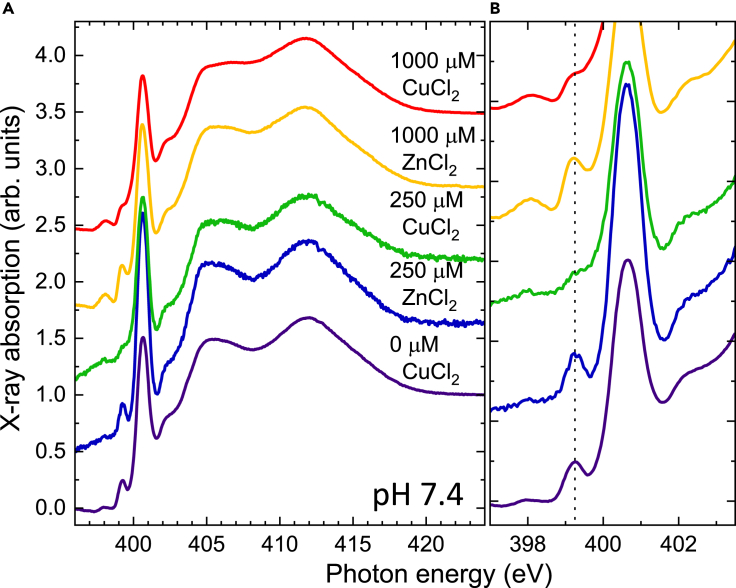
Comparison of the effect of Zn and Cu ions mixed with Aβ_1–40_ at pH 7.4 (A) Comparison of N K-edge spectra with 50 μM Aβ_1–40_ mixed with CuCl_2_ and ZnCl_2_ solutions of different concentrations. A zoom is shown in (B). The ZnCl_2_ sample was prepared by using the same conditions as applied for the CuCl_2_ sample. The dashed vertical line in (B) follows the pre-edge feature discussed in the main text.

**Figure 4:**
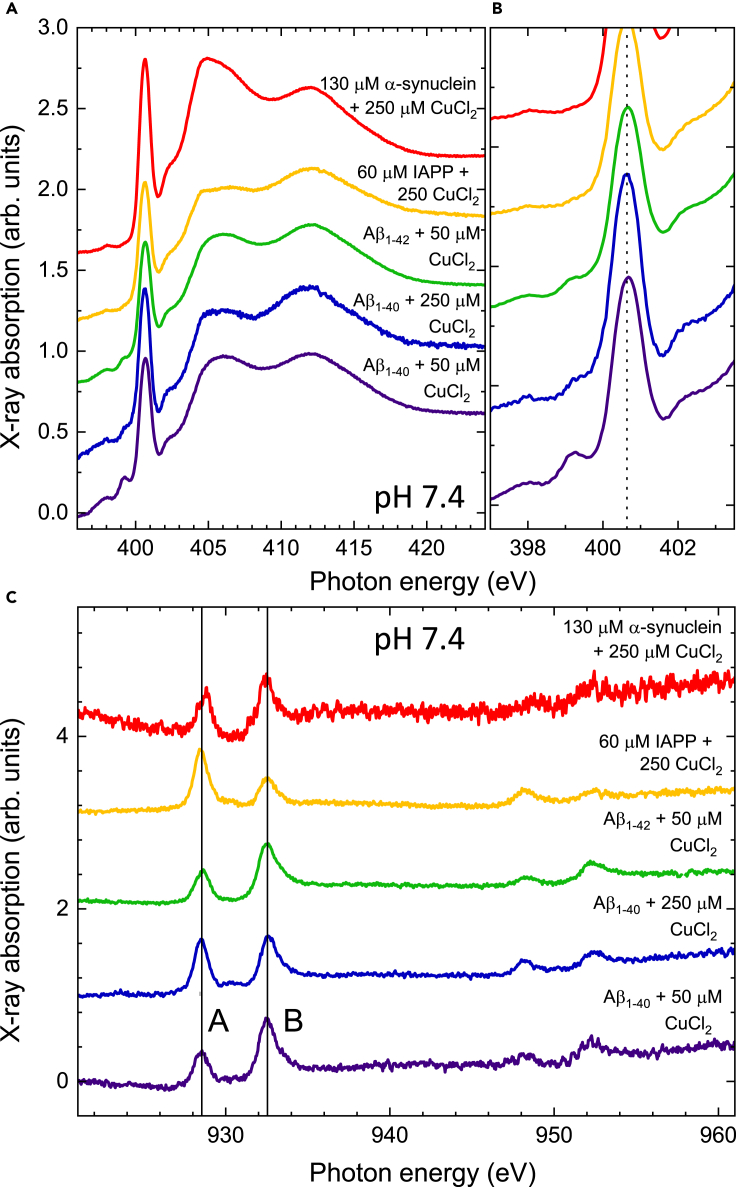
Comparison of the effect of CuCl_2_ mixed with different peptides at pH 7.4 (A and B) N K-edge and (C) Cu L_2,3_-edge spectra recorded on different peptides prepared with CuCl_2_ as indicated in the plot. All samples were prepared in 20 mMKPi buffer, at pH 7.4 by varying the concentrations of CuCl_2_. Aβ_1-40_, Aβ_1-42_, IAPP, and α-Syn were prepared at the concentrations of 50 μM, 50 μM, 60 μM, and 130 μM, respectively. Vertical dashed and solid lines in (B) and (C) follow the features discussed in the main text.

In Figure 3, the N K-edge absorption spectra of Aβ_1-40_ mixed with CuCl_2_ or ZnCl_2_ solutions at different concentrations are plotted. It is striking that independent of the concentration the pre-peak features are essentially unchanged in the presence of ZnCl_2_, while they disappear almost completely with CuCl_2_. This indicates that the interaction of the Aβ_1-40_ peptide with Cu is entirely different from the one with Zn. Furthermore, it confirms that the pre-peak feature of the N K-edge spectra is indeed a unique fingerprint of Aβ_1-40_ upon binding to Cu. We also investigated the absorption spectra of Cu with Parkinson-associated α-syn, type II diabetes-related IAPP, or Aβ_1-42_. Figure 4 displays N K-edge and Cu L-edge absorption spectra of different peptides/proteins mixed with CuCl_2_. In comparison with Aβ_1-40_, Aβ_1-42_ exhibits a slightly higher **B**/**A** ratio of the L_3_-edge at a concentration of 50 μM. The difference could indicate a different reaction of Aβ_1-40_ and Aβ_1-42_ to Cu(II) in the redox cycling. In the presence of other amyloid proteins, we also observed an increased ratio of **B**/**A** in the L_3_-edge, suggesting the existence of the redox cycling. As their sequences vary, each protein may display a distinct mechanism of redox cycling which is beyond the scope of this study.

## Discussion

Briefly, by using N K-edge and Cu L_2,3_ soft X-ray absorption spectroscopy, we simultaneously characterize the Cu and N atoms in the Aβ-Cu complex. The pre-peak feature in the N K-edge spectra of Aβ_1-40_ at 399.3 eV disappears upon binding to CuCl_2_ in contrast to the case of ZnCl_2_. We attribute the loss of this pre-peak feature to the interaction of Aβ_1-40_ with CuCl_2_. The distinguished binding feature of Aβ to Cu(II) and Zn(II) can be caused by the different N-terminal amine coordination of Aβ to these metal ions (Atrián-Blasco et al., 2017). Compared to the Cu binding mentioned in the introduction, the Zn(II) ion interacts with His6 and His13/His14, and with two carboxylate residues from Glu11 and Asp1 or Glu3 or Asp7 rather than with the N-terminal amine (Alies et al., 2016; Atrián-Blasco et al., 2017; Faller et al., 2014). We therefore believe that the N-terminal amine contributes to the pre-peak feature in the N K-edge spectra. Our observation also supports a situation similar to what is observed by circular dichroism (CD) by which the more potent β-sheet structure and extensive aggregation can be induced by Cu but not by Zn (Ghalebani et al., 2012). The loss of the pre-peak feature might be caused by a change of the valence state toward Cu(I) of the peptide-coordinated Cu ions. Alternatively, a structural modification through the Cu-Aβ_1-40_ hybridization leading to more buried Cu(I) centers in conjunction with the surface-sensitive (∼5 nm) X-ray detection could lead to a decrease of the **B**/**A** ratio. On the contrary, zinc acts as a chaperone to reduce the fibril formation (Abelein et al., 2015) and therefore retains the pre-peak feature.

Our results are in agreement with previous EPR studies in which Cu(II)/Aβ_1-16_ complexes possess distinct redox behavior (Trujano-ortiz and Quintanar, 2015), indicative of an intermediate Cu(I) species. With the peak **B** of the Cu L_2,3_ spectra, we observe a significant conversion toward monovalent Cu(I) from Cu(II) after interacting with Aβ. The presence of Cu(I) in the Aβ_1-40_-Cu complex shown in Figures 1C and 2C is a straightforward evidence of the aforementioned interaction. Cu(I) can originate from redox cycling of the Cu(II)-Aβ complex, in which three possible intermediate states of bis-His(Aβ)-Cu(I) are involved (Faller et al., 2014)(Trujano-ortiz and Quintanar, 2015). The recycling starts with geometrical reorganization after Cu(II) leaves the N-terminal and the carbonyl groups, and then a bis-His(Aβ)-Cu(I) complex is formed (Faller et al., 2014)(Trujano-ortiz and Quintanar, 2015). The pH may influence the recycling by changing the protonation of His. This is further supported by the pH dependence of the Cu spectra where we reveal the relevant physical chemistry linking acidosis and Alzheimer's disease. As revealed by Table S1, in Cu-Aβ aggregates, the Cu(I) peak (peak **B**/**A** ratio) is much less significant at a pH of 5.5 as compared with pH 7.4, presumably caused by faster aggregation of Cu-Aβ at pH 5.5 than pH 7.4 (Ghalebani et al., 2012). These insoluble Aβ aggregates with Cu(II) at pH 5.5 may be less accessible for the reduction of Cu(II) than the ones at pH 7.4. In conclusion, our soft X-ray study reveals the physical chemistry of the Cu and N atoms behind the Aβ_1-40_ and copper interaction at varied conditions mimicking the acidosis in Alzheimer's disease.

### Limitations of the study

The study demonstrates the interaction of amyloid-beta with copper and zinc using synchrotron-based soft X-ray absorption spectroscopy. One of the limitations is that the zinc atoms are spectroscopically less accessible because of their weaker X-ray absorption due to the absence of strong resonant features. The interaction of amyloid-beta with zinc can thus only be observed indirectly through the nitrogen K-edge spectroscopy. Another limitation is the spatial resolution, therefore no statements can be made about the spatial distribution of the copper ions and, for example, about its homogeneity. In the present study, spatial resolution is limited by the rather large X-ray spot size on the order of 0.5 mm^2^. In the future, spatially resolved experiments will be performed using suitable X-ray microscopes.

## STAR★Methods

### Key resources table

**Table:** 

REAGENT or RESOURCE	SOURCE	IDENTIFIER
**Chemicals, peptides, recombinant proteins**		

Recombinant amyloid-beta 1-40	AlexoTech AB, Umea, Sweden	AB-100-10
Recombinant amyloid-beta 1-42	rPeptide	A-1163-1
CuCl_2_	Sigma-Aldrich	451,665
ZnCl_2_	Sigma-Aldrich	208,086
Amylin (1–37)	AnaSpec	AS-60804

**Bacterial and virus strains**		

E.coliBL21 (DE3) pLysS strain	ThermoFisher	C606010

**Deposited data**		

Raw and analyzed data	This paper	NA

**Software and algorithms**		

Origin	Originlab	https://www.originlab.com

### Resource availability

#### Lead contact

Further information and requests for resources and reagents should be directed to and will be fulfilled by the lead contacts Jinghui Luo (jinghui.luo@psi.ch) and Jan Dreiser (jan.dreiser@psi.ch).

#### Materials availability

This study did not generate new unique reagents.

### Method details

#### Sample preparation

Aβ_1-40_ and Aβ_1-42_ peptides were bought from AlexoTechAB(Cat.ID: AB-100-10) and rPeptide (Cat.ID: A-1163-1), respectively. Both peptides were dissolved in 10 mMNaOH at a concentration of 1 mg/mL and sonicated in a water-ice bath for 1 min. Afterward, it was diluted for the preparation with the Aβ and CuCl_2_/ZnCl_2_ complex. CuCl_2_ (Cat. ID: 451,665) and ZnCl_2_ (Cat.ID: 208,086) were ordered from Sigma-Aldrich. In addition, Amylin (1–37)/IAPP was ordered from AnaSpec (Cat.ID: AS-60804) and dissolved to 20 mMKPi buffer after evaporating HFIP (Hexafluoroisopropanol). α-Synuclein proteins were expressed and purified followed by the established protocols (Huang et al., 2005). Briefly, *E.coli*BL21 (DE3) pLysS strain was used to express α-Synuclein and induced with 0.5 mMIPTG for 5 h at 37°. Sucrose osmotic shock was applied for releasing α-Synuclein from the periplasm. Ion-exchange and size-exclusion chromatography were then used for further purification of α-Synuclein. The fractions with the purified protein were verified by running SDS page. 20 mMKPi buffer was used for preparing/diluting samples for the measurements. Regarding the incubated samples in Table S1, they were prepared with the highest concentration of 1000 μM of CuCl_2_ in 200 μL KPi buffer, and then incubated under quiescent condition for 4 h at room temperature or for 1 day at 30°C. Others samples mentioned in the manuscript were prepared in 200 μL, 20mMKPi buffer (pH 5.5 and pH 7.4) with 0, 10, 50, 250 or 1000 μM CuCl_2_/ZnCl_2_, 50 μL of which were used to deposit for 10 min on the surface of a silicon dioxide wafer. All of the deposited samples were immediately dried with nitrogen gas for X-ray absorption measurements.

#### X-ray absorption spectroscopy

Spectra were recorded at the X-Treme beamline (Piamonteze et al., 2012) at the Swiss Light Source, Paul Scherrer Institut. To protect the samples from beam damage, an attenuated photon flux and a defocused X-ray spot (∼0.5 mm^2^) on the sample were chosen. Specifically, the impinging X-ray photon flux per area was 0.06 photons/sec/nm^2^ at the Cu L_2,3__-_edges and 0.15 photons/sec/nm^2^ at the N K-edge. No changes of the spectral weight of peaks **A**, **B** over timescales of several tens of minutes could be observed. Unless explicitly stated otherwise, the spectra were recorded at room temperature in normal incidence of the X-ray beam. The Cu L_2,3_ spectra were normalized for the sum of the peak areas **A** and **B** to yield a constant of 1.0 after subtracting the base line. Furthermore, the N K-edge spectra were base line subtracted and normalized by a linear scaling such that the post edge was forced to 1. Reference spectra at the Cu L_2,3_-edges and at the N K-edge were obtained on a drop cast film of Cu(II)-pthalocyanine on silicon dioxide (Figure S2).

## Data Availability

All data reported in this paper will be shared by the lead contacts Jinghui Luo (jinghui.luo@psi.ch) and Jan Dreiser (jan.dreiser@psi.ch) upon request. This paper does not report original code. Any additional information required to reanalyze the data reported in this paper is available from the lead contacts Jinghui Luo (jinghui.luo@psi.ch) for the samples and Jan Dreiser (jan.dreiser@psi.ch) for the X-ray measurements upon request.

## Footnotes

Supplemental information can be found online at https://doi.org/10.1016/j.isci.2021.103465.

## References

[bib1] Abelein A., Gräslund A., Danielsson J. (2015). Zinc as chaperone-mimicking agent for retardation of amyloid β peptide fibril formation. Proc. Natl. Acad. Sci. U S A.

[bib2] Alí-Torres J., Maréchal J.-D., Rodríguez-Santiago L., Sodupe M. (2011). Three dimensional models of Cu(2+)-Aβ(1-16) complexes from computational approaches. J. Am. Chem. Soc..

[bib3] Alies B., Conte-Daban A., Sayen S., Collin F., Kieffer I., Guillon E., Faller P., Hureau C. (2016). Zinc(II) binding site to the amyloid-β peptide: Insights from spectroscopic studies with a wide series of modified peptides. Inorg. Chem..

[bib4] Atrián-Blasco E., Conte-Daban A., Hureau C. (2017). Mutual interference of Cu and Zn ions in Alzheimer’s disease: Perspectives at the molecular level. Dalt. Trans..

[bib5] Broersen K., Jonckheere W., Rozenski J., Vandersteen A., Pauwels K., Pastore A., Rousseau F., Schymkowitz J. (2011). A standardized and biocompatible preparation of aggregate-free amyloid beta peptide for biophysical and biological studies of Alzheimer’s disease. Protein Eng. Des. Sel..

[bib6] Dear A.J., Meisl G., Šarić A., Michaels T.C.T., Kjaergaard M., Linse S., Knowles T.P.J. (2020). Identification of on- And off-pathway oligomers in amyloid fibril formation. Chem. Sci..

[bib7] Dong J., Atwood C.S., Anderson V.E., Siedlak S.L., Smith M.A., Perry G., Carey P.R. (2003). Metal binding and oxidation of amyloid-β within isolated senile plaque cores: Raman microscopic evidence. Biochemistry.

[bib8] Dorlet P., Gambarelli S., Faller P., Hureau C. (2009). Pulse EPR spectroscopy reveals the coordination sphere of copper(II) ions in the 1-16 amyloid-β peptide: A key role of the first two N-terminus residues. Angew.Chem..

[bib9] Drew S.C., Masters C.L., Barnham K.J. (2009). Alanine-2 carbonyl is an oxygen ligand in Cu^2+^ coordination of Alzheimer’s disease amyloid-β peptide - Relevance to N-terminally truncated forms. J. Am. Chem. Soc..

[bib10] Drew S.C., Noble C.J., Masters C.L., Hanson G.R., Barnham K.J. (2009). Pleomorphic copper coordination by Alzheimer’s disease amyloid-β peptide. J. Am. Chem. Soc..

[bib11] Ehrnhoefer D.E., Bieschke J., Boeddrich A., Herbst M., Masino L., Lurz R., Engemann S., Pastore A., Wanker E.E. (2008). EGCG redirects amyloidogenic polypeptides into unstructured, off-pathway oligomers. Nat. Struct. Mol. Biol..

[bib12] Faller P., Hureau C., La Penna G. (2014). Metal ions and intrinsically disordered proteins and peptides: From Cu/Zn amyloid-β to general principles. Acc. Chem. Res..

[bib13] Ghalebani L., Wahlström A., Danielsson J., Wärmländer S.K.T.S., Gräslund A. (2012). pH-dependence of the specific binding of Cu(II) and Zn(II) ions to the amyloid-β peptide. Biochem. Biophys. Res. Commun..

[bib14] Glabe C.G. (2008). Structural classification of toxic amyloid oligomers. J. Biol. Chem..

[bib15] Grioni M., van Acker J.F., MT Czyzyk J.F. (1992). Unoccupied electronic structure and core-hole effects in the x-ray-absorption spectra of Cu_2_O. Phys. Rev. B.

[bib16] Han J., Lee H.J., Kim K.Y., Nam G., Chae J., Lim M.H. (2021). Mechanistic approaches for chemically modifying the coordination sphere of copper–amyloid-β complexes. Proc. Natl. Acad. Sci. U. S. A.

[bib17] Hatsui T., Yamamoto T., Tajima H., Kosugi N. (2004). Cu L_2,3_-edge X-ray absorption spectra of (2,5-dimethyl-N,N′-dicyanoquinonediimine)_2_Li_1-x_Cu_x_ alloys. Chem. Phys..

[bib18] Huang C., Ren G., Zhou H., Wang C. (2005). A new method for purification of recombinant human alpha-synuclein in Escherichia coli. Protein Expression Purif..

[bib19] Ittner L.M., Götz J. (2011). Amyloid-β and tau--a toxic pas de deux in Alzheimer’s disease. Nat. Rev. Neurosci..

[bib20] Karr J.W., Kaupp L.J., Szalai V.A. (2004). Amyloid-β binds Cu^2+^ in a mononuclear metal ion binding site. J. Am. Chem. Soc..

[bib21] Kenche V.B., Barnham K.J. (2011). Alzheimer’s disease & metals: therapeutic opportunities. Br. J. Pharmacol..

[bib22] Kvashnina K.O., Butorin S.M., Modin A., Werme L., Nordgren J., Guo J.H., Berger R. (2009). Electronic structure of complex copper systems probed by resonant inelastic X-ray scattering at Cu L_3_ edge. Physica B.

[bib23] Leinweber P., Kruse J., Walley F.L., Gillespie A., Eckhardt K.U., Blyth R.I.R., Regier T. (2007). Nitrogen K-edge XANES - an overview of reference compounds used to identify “unknown” organic nitrogen in environmental samples. J. Synchrotron Radiat..

[bib24] Luo J., Yu C.-H., Yu H., Borstnar R., Kamerlin S.C.L., Gräslund A., Abrahams J.P., Wärmländer S.K.T.S. (2013). Cellular polyamines promote amyloid-Beta (aβ) Peptide fibrillation and modulate the aggregation pathways. ACS Chem. Neurosci..

[bib25] Luo J., Wärmländer S.K.T.S., Gräslund A., Abrahams J.P. (2014). Alzheimer peptides aggregate into transient nanoglobules that nucleate fibrils. Biochemistry.

[bib26] Maynard C.J., Bush A.I., Masters C.L., Cappai R., Li Q.-X. (2005). Metals and amyloid-beta in Alzheimer’s disease. Int. J. Exp. Pathol..

[bib27] Mold M., Ouro-Gnao L., Wieckowski B.M., Exley C. (2013). Copper prevents amyloid-β_1-42_ from forming amyloid fibrils under near-physiological conditions in vitro. Sci. Rep..

[bib28] Piamonteze C., Flechsig U., Rusponi S., Dreiser J., Heidler J., Schmidt M., Wetter R., Calvi M., Schmidt T., Pruchova H. (2012). X-Tremebeamline at SLS: X-ray magnetic circular and linear dichroism at high field and low temperature. J. Synchrotron Radiat..

[bib29] Pithadia A.S., Lim M.H. (2012). Metal-associated amyloid-β species in Alzheimer’s disease. Curr.Opin. Chem. Biol..

[bib30] Posadas Y., Parra-Ojeda L., Perez-Cruz C., Quintanar L. (2021). Amyloid β perturbs Cu(II) binding to the prion protein in a site-specific manner: Insights into its Potential neurotoxic mechanisms. Inorg. Chem..

[bib31] Selkoe D.J., Hardy J. (2016). The amyloid hypothesis of Alzheimer’s disease at 25 years. EMBO Mol. Med..

[bib32] Shearer J., Callan P.E., Tran T., Szalai V.A. (2010). Cu K-edge X-ray absorption spectroscopy reveals differential copper coordination within amyloid-β oligomers compared to amyloid-β monomers. Chem. Commun..

[bib33] Shimizu K.I., Maeshima H., Yoshida H., Satsuma A., Hattori T. (2001). Ligand field effect on the chemical shift in XANES spectra of Cu(II) compounds. Phys. Chem. Chem. Phys..

[bib34] Streltsov V.A., Titmuss S.J., Epa V.C., Barnham K.J., Masters C.L., Varghese J.N. (2008). The structure of the amyloid-β peptide high-affinity copper II binding site in Alzheimer disease. Biophys. J..

[bib35] Tiiman A., Luo J., Wallin C., Olsson L., Lindgren J., Jarvet J., Roos P., Sholts S.B., Rahimipour S., Abrahams J.P. (2016). Specific binding of Cu(II) ions to amyloid-beta peptides bound to aggregation-inhibiting molecules or SDS micelles creates complexes that generate radical oxygen species. J. Alzheimer’s Dis..

[bib36] Trujano-ortiz L.G., Quintanar L. (2015). Redox cycling of copper−amyloid β 1−16 peptide complexes is highly dependent on the coordination mode. Inorg. Chem..

[bib37] Viles J.H. (2012). Metal ions and amyloid fiber formation in neurodegenerative diseases. Copper, zinc and iron in Alzheimer’s, Parkinson’s and prion diseases. Coord. Chem. Rev..

[bib38] Wu J., Cao C., Loch R.A., Tiiman A., Luo J. (2020). Single-molecule studies of amyloid proteins: From biophysical properties to diagnostic perspectives. Q. Rev. Biophys..

[bib39] Wu J., Blum T.B., Farrell D.P., DiMaio F., Abrahams J.P., Luo J. (2021). Cryo-electron microscopy imaging of Alzheimer’s amyloid-beta 42 oligomer displayed on a functionally and structurally relevant scaffold. Angew. Chem. Int. Ed..

