# Supplementary material for: Amyloid-beta–copper interaction studied by simultaneous nitrogen K and copper L2,3-edge soft X-ray absorption spectroscopy
Source: iScience. 2021 Nov 16;24(12):103465. doi: 10.1016/j.isci.2021.103465 (PMC8710549; doi:10.1016/j.isci.2021.103465)
Supplement: Document S1.Figures S1 and S2 and Table S1 [file mmc1.pdf]

iScience, Volume *24*

Supplemental information

Amyloid-beta–copper interaction studied by simultaneous nitrogen K and copper $L_{2,3}$-edge soft X-ray absorption spectroscopy

Jinghui Luo, Hongzhi Wang, Jinming Wu, Vladyslav Romankov, Niéli Daffé, and Jan Dreiser

SUPPLEMENTAL INFORMATION

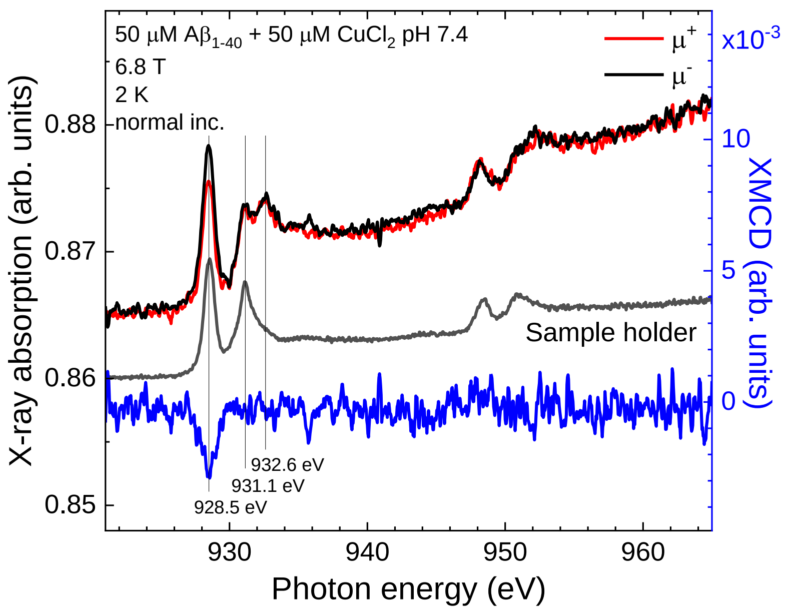

Figure S1. XAS and X-ray magnetic circular dichroism (XMCD) spectra recorded at the Cu $L_{2,3}$ edges. The measurement was conducted at a temperature of 2 K and a magnetic field of 6.8 T on a drop cast sample of 50 μM $A\beta_{1\text{-}40}$ mixed with 50 μM $CuCl_2$ at pH 7.4 The XAS recorded on the sample holder is also shown for comparison. The sample holder spectrum allows to identify the peak appearing at 931.1 eV to originate from the Cu-containing sample holder. In the $A\beta_{1\text{-}40}$ sample this peak also appears because the sample holder was partially hit by the X-ray beam due to a slight misalignment. The XMCD spectrum calculated as the difference between the circularly polarized XAS (μ^+ - μ^-) is shown in blue. An XMCD response is only seen at a photon energy of 928.5 eV coinciding with the first feature [Cu(II)] in the XAS. The absence of XMCD underpins that the other two peaks at a higher photon energy indeed originate from diamagnetic Cu species, i.e., Cu(I) having a complete d-shell.

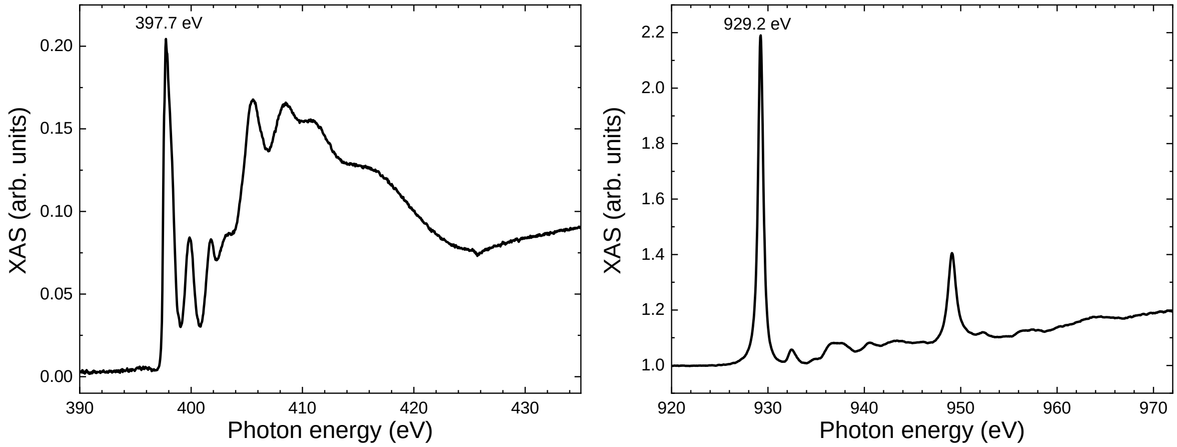

Figure S2. X-ray absorption spectra at the (left) N K-edge and (right) Cu $L_{2,3}$-edges recorded on Cu(II)-phthalocyanine reference samples at room temperature.

Table S1. Peak ratios **B/A** of the Cu L_3 edge at different sample preparation conditions, related to figures 1-3. The error on the peak ratio is estimated to be on the order of 10 percent of the actual value.

Sample	pH	Incubation time	Incubation temperature	Peak ratio B/A
$A\beta_{1-40}$ + 10 µM $CuCl_2$	5.5	None	n/a	1.3
$A\beta_{1-40}$ + 50 µM $CuCl_2$	5.5	None	n/a	0.5
$A\beta_{1-40}$ + 250 µM $CuCl_2$	5.5	None	n/a	0.1
$A\beta_{1-40}$ + 1000 µM $CuCl_2$	5.5	4 h	Room temperature	0.04
$A\beta_{1-40}$ + 1000 µM $CuCl_2$	5.5	1 d	30 °C	0.1
$A\beta_{1-40}$ + 50 µM $CuCl_2$	7.4	None	n/a	1.7
$A\beta_{1-40}$ + 250 µM $CuCl_2$	7.4	None	n/a	0.8
$A\beta_{1-40}$ + 1000 µM $CuCl_2$	7.4	4 h	Room temperature	0.4
$A\beta_{1-40}$ + 1000 µM $CuCl_2$	7.4	1 d	30 °C	0.27
